# Virus Encoded MHC-Like Decoys Diversify the Inhibitory KIR Repertoire

**DOI:** 10.1371/journal.pcbi.1003264

**Published:** 2013-10-10

**Authors:** Paola Carrillo-Bustamante, Can Keşmir, Rob J. de Boer

**Affiliations:** Theoretical Biology & Bioinformatics, Department of Biology, Utrecht University, Utrecht, The Netherlands; Imperial College London, United Kingdom

## Abstract

Natural killer (NK) cells are circulating lymphocytes that play an important role in the control of viral infections and tumors. Their functions are regulated by several activating and inhibitory receptors. A subset of these receptors in human NK cells are the killer immunoglobulin-like receptors (KIRs), which interact with the highly polymorphic MHC class I molecules. One important function of NK cells is to detect cells that have down-regulated MHC expression (missing-self). Because MHC molecules have non polymorphic regions, their expression could have been monitored with a limited set of monomorphic receptors. Surprisingly, the KIR family has a remarkable genetic diversity, the function of which remains poorly understood. The mouse cytomegalovirus (MCMV) is able to evade NK cell responses by coding “decoy” molecules that mimic MHC class I. This interaction was suggested to have driven the evolution of novel NK cell receptors. Inspired by the MCMV system, we develop an agent-based model of a host population infected with viruses that are able to evolve MHC down-regulation and decoy molecules. Our simulations show that specific recognition of MHC class I molecules by inhibitory KIRs provides excellent protection against viruses evolving decoys, and that the diversity of inhibitory KIRs will subsequently evolve as a result of the required discrimination between host MHC molecules and decoy molecules.

## Author Summary

Human natural killer (NK) cells patrol peripheral tissue, monitoring changes on the surface of body cells. They express a network of activating and inhibitory receptors called the killer immunoglobulin-like receptors (KIRs). The main ligands of inhibitory KIRs are MHC class I molecules, which present viral peptides to other immune cells. Several herpes viruses interfere with MHC expression, and when a virus down-regulates MHC class I, NK cells loose an inhibitory signal, become activated and kill the infected cell. The KIR family has a large genetic diversity. However, for the recognition of “missing” MHC molecules this diversity seems redundant as one set of receptors should be sufficient. To study why the KIR system has evolved such a high complexity, we developed an *in-silico* model, simulating the evolution of populations infected with a herpes-like virus. Next to down regulating MHC-I molecules, these viruses are able to escape the NK cell response by expressing MHC-decoys engaging the inhibitory KIRs. We show that specific KIR-MHC interactions protect against viruses expressing decoys. Because of the provided protection, specific inhibitory KIRs have an evolutionary advantage, giving rise to a high level of diversity. We propose that herpes-like viruses evolving decoys affect in the evolution of KIRs.

## Introduction

Natural killer (NK) cells constitute 5–25% of the lymphocytes circulating in human peripheral blood [1]. Being part of the innate immune response, they play an important role in the defense against viral infections and in tumor surveillance [2]. In contrast to T and B cells, NK cells do not use somatic gene rearrangements to generate a diverse repertoire of cells expressing unique receptors. Instead, they sample a subset of receptors from a repertoire of activating and inhibitory receptors encoded by the germline.

Individual NK cells express several inhibitory and activating receptors that recognize, among others, major histocompatibility complex (MHC) class I and MHC class I related molecules as their ligands [3]. The interaction between these receptors and ligands generates signals that either allow the NK cell to attack target cells or prevent it from harming healthy tissue. Several viruses down-regulate the expression of host MHC class I molecules, and since these molecules are often inhibitory ligands of NK cell receptors, loss of their expression on the infected cell induces NK cell activation. This mechanism by which NK cells attack MHC-class I deficient cells was coined by Kärre et. al [4] as “missing-self” detection.

In humans there are two main receptor families contributing to missing-self detection. The inhibitory receptor CD94/NKG2A binds to complexes of the human leukocyte antigen (HLA)-E, presenting peptides derived from the leader sequences of HLA-A, -B, and -C [5], [6]. In this inhibitory interaction both receptor and ligand are highly conserved, and the down-stream effects are remarkably similar in different individuals [7]. In contrast, killer immunoglobulin-like receptors (KIR), recognizing the highly polymorphic HLA-A, -B, and -C molecules, can be both inhibiting and activating, are very diverse, and rapidly evolving [8]. Engagement of either inhibitory KIR or NKG2A inhibits the activity of an NK cell, preventing target cell lysis. Phylogenetic studies have shown that the CD94/NKG2 system is relatively old, and that the KIR genes have evolved more recently [9]. Thus, there are two NK cell receptor systems, one conserved and one highly diverse, detecting abnormalities in MHC expression on cell surfaces.

KIRs are encoded by a large family of genes exhibiting a remarkable variability in gene content and allelic polymorphism. The KIR complex in humans contains up to 14 KIR genes and pseudogenes [10] that are arranged into two main groups of haplotypes, A and B, differing in size, gene content, function, and disease association [11]. Since the MHC and KIR loci are on different chromosomes (in humans, on chromosome 6 and 19, respectively), a tremendous number of possible receptor-ligand combinations exists on the population level. Moreover, KIR-HLA interactions are rather specific, with four mutually exclusive epitopes on HLA molecules (A3/11, Bw4, C1 and C2) so far identified as inhibitory KIR ligands [12]. KIR interactions with HLA-C are sensitive to polymorphisms at distal positions [13] and to bound peptides [14], affecting KIR binding, and with that the functionality of NK cells. It is widely accepted that the fine specificity and vast diversity of B and T cell receptors per individual render each host the capacity to recognize many different pathogens, and to distinguish them from healthy tissue. But how does the specificity and much smaller diversity of NK cell receptors per individual contribute to the host's survival? If missing-self detection were the main function of inhibitory KIRs, and since this can also be achieved by the conserved receptor NKG2A, why have these more recent NK cell receptors evolved to become specific, polymorphic, and diverse?

Specific KIR alleles have been associated with particular infections such as HIV, HCV, cerebral malaria, and with several pregnancy disorders [15]–[21]. Indeed, population genetic studies have suggested that a high degree of KIR diversity is necessary for surviving epidemic infections and population bottlenecks [22], but no explicit evolutionary mechanism selecting for novel KIR alleles has been proposed so far. Why polymorphic KIRs would be required to just detect MHC down-regulation remains puzzling.

Cytomegaloviruses (CMV) and other viruses from the herpes family have large genomes that encode for a series of immuno-evasive mechanisms, targetting key molecular steps necessary for a successful immune response [23]–[25]. Particularly important for the evasion of NK cell surveillance are MHC-I like molecules that can engage inhibitory NK cell receptors, like the mouse CMV (MCMV) encoded glycoprotein m157 binding to Ly49 receptors [26], [27], and the human CMV (HCMV) UL18 engaging the inhibitory leukocyte immunoglobulin-like receptor LIR–1 [28]. Not all of these evasion strategies have been elucidated yet, and it remains unclear whether m157 and UL18 are the only decoy molecules evolved by herpes viruses. Recent studies have revealed a strong imprint in the KIR repertoire of CMV seropositive individuals [29], [30], suggesting that additional CMV evasion mechanisms interacting directly with KIRs (e.g. novel decoy molecules yet to be identified) exert a strong selection pressure.

We investigated whether the presence of viral decoys like MCMV m157 or HCMV UL18 can drive the expansion of specific, inhibitory NK cell receptors, such as KIRs. We performed our study with an agent-based computer model of co-evolving hosts and viruses. Our results show that specific MHC recognition by inhibitory KIRs provides excellent protection against viruses evolving decoy molecules, and that diversity in the receptor system can be a consequence of this specific interaction between MHC and KIR molecules.

## Results

To investigate the evolution of the KIR genes we developed an agent-based model consisting of a host population infected with a non-lethal herpes-like virus causing chronic infections. In this model, individuals were randomly selected during every time step to be confronted with one of the randomly chosen events: birth, viral infection, and death. We modeled a host population of simplified humans carrying one MHC locus and one KIR haplotype composed of five genes. Here, we only modeled inhibitory KIRs and work on activating receptors is in progress. All hosts were initialized with the same randomly generated KIR haplotype, but with different MHC genes. While we allowed for mutation of novel KIR genes during the birth event, mutation of MHC molecules was not considered, and the initial MHC polymorphism (14 alleles, mimicking the number of common HLA–C molecules) remained constant throughout the simulation. Although the host population considered here is inspired on humans, the model remains simple to allow it to be general and also apply to the evolution of KIR in ‘higher’ primates in which the KIR genes also expanded and diversified [31].

**Figure 1:**
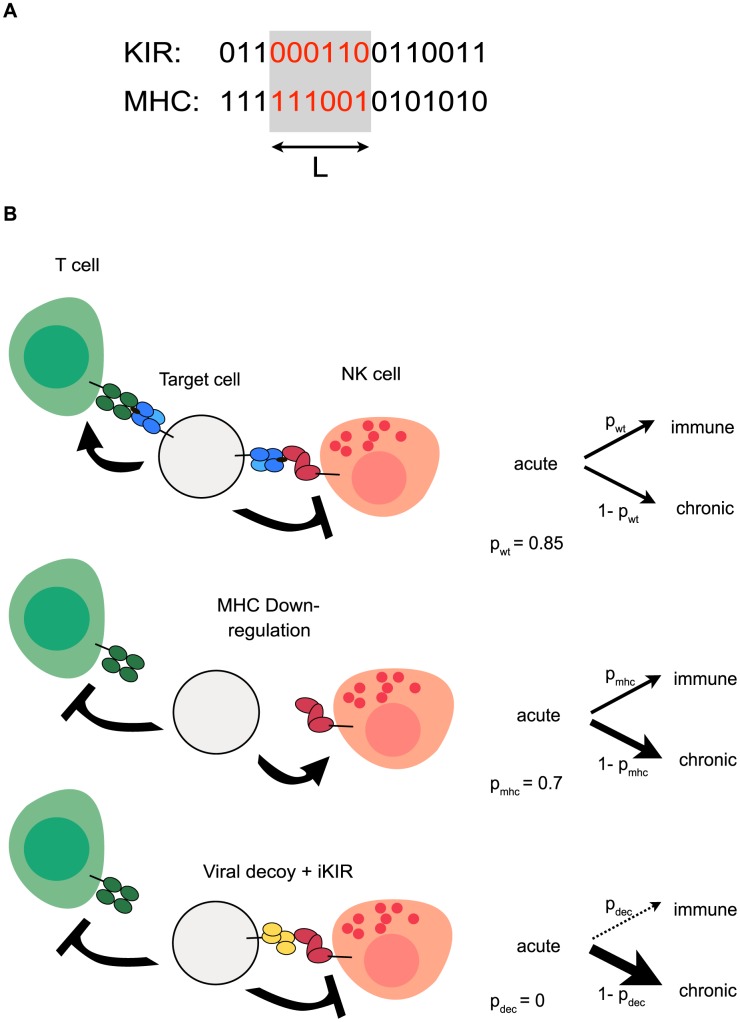
Schematic model description. (A) KIRs and MHC molecules are implemented as bitstrings. The receptor-ligand recognition is modeled via complementary matching. Here, the longest adjacent match is 6 bits long, and when the length of this match exceeds the threshold 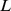, the MHC molecules can be recognized by the KIR. (B) Description of the viral evasion mechanisms. The wild type virus can evolve MHC-downregulation and a decoy ligand for KIRs. The immune escape of the virus after evolution of the immunoevasive mechanisms is modeled by decreasing the clearance probability 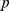. The arrow-headed lines indicate the activation of the T cell or the NK cell, whereas the bar-headed lines indicate their inhibition.

KIR, MHC, and viral MHC decoys were modeled with bit strings (i.e., randomly generated sequences of zero and ones), as a simplified representation of amino acid sequences (see Material and Methods). Receptor-ligand binding depended on the longest adjacent complementary match between their bit strings (Fig. 1 A). If the length of the longest match reached a threshold 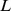, the molecules could interact. Table 1 depicts the relation between the specificity, i.e., the likelihood of such an interaction, and 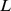.

**Table 1:** Relationship between specificity and crucial parameters for clearing the infection.

Specificity ( 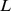 )	MHC recognition ( 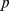 )	Licensed KIRs ( 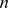 )	Protection ( 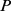 )	Heterozygous advantage ( 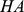 )
1	1.000	10.000	0.000	1.000
2	0.959	9.984	0.000	1.000
3	0.696	9.078	0.000	1.000
4	0.396	6.352	0.041	1.007
5	0.197	3.551	0.458	1.112
6	0.095	1.818	0.833	1.367
7	0.041	0.807	0.966	1.657
8	0.024	0.477	0.988	1.783
9	0.011	0.222	0.998	1.894
10	0.006	0.120	0.999	1.941

The specificity of a KIR molecule 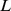 is measured in bits. The analytical expectations of its probability of recognizing MHC, the expected repertoire of licensed KIRs, the expected protection against decoy viruses, and the expected heterozygous advantage of two KIR haplotypes were calculated as described in Material and Methods.

In every host, those KIRs that failed to recognize any of the two MHC molecules present in the individual were deleted from the host's repertoire, leaving each host with only a “licensed” KIR repertoire. Only those KIR molecules that were licensed participated in the immune response, mimicking the MHC-dependent education process during NK cell development [32]. The expected number of licensed KIRs per host, and consequently the probability of the host being protected, depends on the specificity of the KIR molecules, and can be calculated as described in Material and Methods (see Table 1).

**Figure 2:**
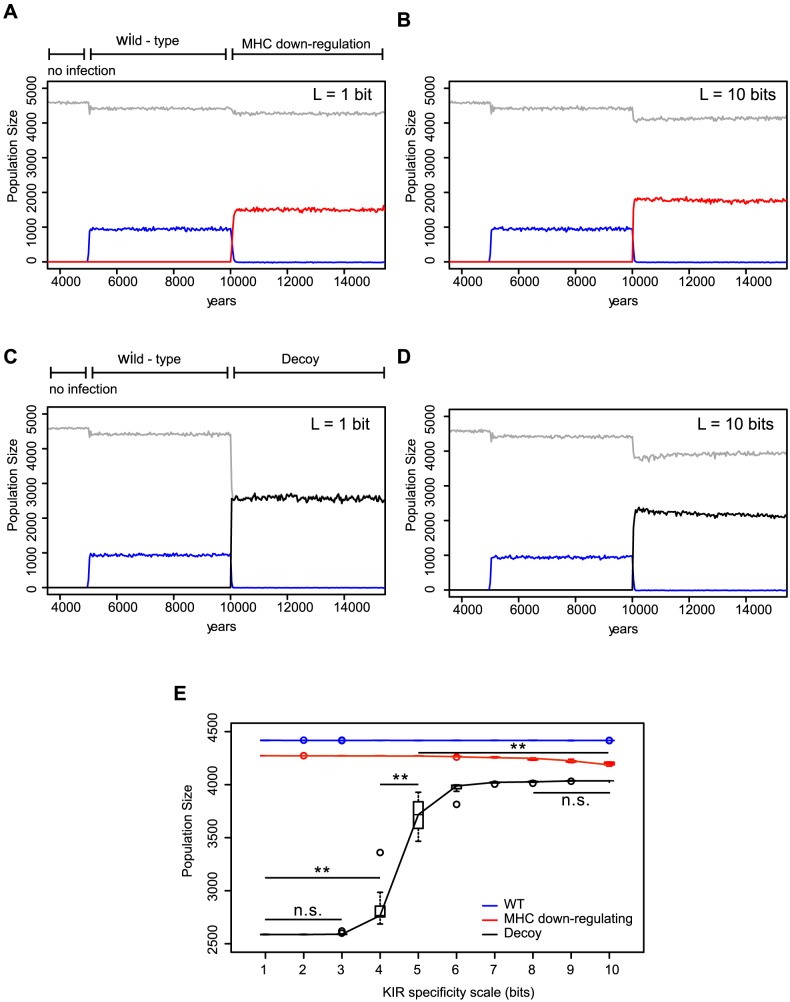
KIR specificity is protective against viruses evolving MHC-like decoys. A host population was inoculated with a wild type virus after a “burn in” period of 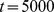 years; we allowed for mutation of the virus 5000 years after the initial epidemic. (A) The MHC down-regulating virus (red line) is fitter than the wild type virus (blue line), infecting more individuals and resulting in a larger decrease of the total population size (grey line). Here, the host population has degenerate KIRs (with 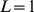 bit). (B) Population with a highly specific receptor-ligand recognition (with 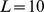 bits). (C) Viruses evolving MHC down-regulation and decoy molecules (black line) take over the wild type virus (blue line), infecting more individuals and resulting in a larger decrease of the total population size (grey line). In a host population with degenerate KIRs, i.e., 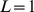 bits, almost all individuals are infected with the virus. (D) The spread of the virus is reduced in host populations with specific KIRs, e.g., 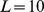 bits. (A)–(D) single representative simulations of the first 15 000 years of the simulations. (E) Ten simulations were carried out per specificity value ranging from 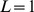 bit to 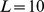 bits. At the end of each simulation (i.e. 200 000 years), the mean of the total population size over the last 100 000 years was taken as a measure of the protection level of the host population. Protection against the wild type virus (blue) was not dependent on the specificity of the KIRs. Protection against an MHC down-regulating virus (red) was slightly, yet significantly worse in simulations with specific KIRs (i.e. 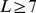), whereas protection against the virus evolving decoy molecules (black line) increased drastically in simulations with specific systems (i.e. 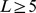). (** represent *p* values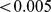 and were calculated using the Mann-Whitney *U* test. The boxes represent the interquartile range, the thick horizontal lines represent the median, and the circles are outliers.).

Infection of a host started with a short acute phase, after which the individual could either recover, or become chronically infected. We considered 16 different viruses: one “wild type” virus, one “MHC down-regulating” virus, and 14 “MHC decoy” viruses (i.e., one for each of the 14 MHC molecules in our model). We did not allow for superinfection and hosts could be infected with one of the 16 viruses only. The evolution of decoy proteins was modeled by allowing the virus to adopt a randomly selected MHC molecule from its host. Therefore, each decoy protein was actually an MHC molecule. The host populations were first inoculated with the “wild-type” virus, which was typically cleared after the acute phase because of the assumed immune response of both, cytotoxic T and NK cells (Fig. 1 B). The effect of the immune response was modeled by a parameter describing the probability of clearing the infection. For the wild-type virus, this parameter was set relatively high, i.e. 85% of the wild type infections were cleared, resulting in approximately 30% of the individuals becoming chronically infected (Fig. 1 B, and Fig. 2 blue line). The clearance probability was lower for the down-regulating and decoy viruses, resembling their immune escape. Viruses establishing chronic infections spread over much longer periods of time than those that do not; and we therefore expect viruses capable of MHC down-regulation and carrying decoy molecules to outcompete wild-type viruses. We used this model to investigate the evolution of the KIR system, and compared the selection pressures imposed by different viral variants.

### Specific inhibitory KIRs are disadvantageous in populations infected with a virus down-regulating MHC expression

If detection of MHC class I is the main function of inhibitory KIRs, we expect that KIRs do not need to be specific nor diverse because missing self detection can be achieved by a limited set of monomorphic receptors. To address this, we analyzed the effects of KIR specificity on populations infected with a virus that is capable of MHC down-regulation to escape T cell response. The immune escape of this mutant was modeled by decreasing the probability of clearing the infection (from 85% to 70%), resulting in a better spread of the virus and a larger fraction of individuals becoming chronically infected (Fig. 1 B, and Fig. 2 A red line).

We screened the average population size as an indicator of the hosts' protection after an infection. By comparing simulations with degenerate KIRs with those with specific KIRs, we observed significant differences in population sizes (from 4300 in 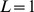 to 4100 in 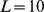, 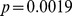, Mann-Whitney *U* test). Although the effect of KIR specificity on protection against an MHC down-regulating virus was small, it clearly indicated that hosts with highly specific KIR–MHC interactions were more vulnerable than those having degenerate KIRs (Fig. 2 A,B,E). Why is a high KIR specificity disadvantageous during an infection with an MHC down-regulating virus? Since degenerate KIRs (i.e. 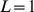) are likely to recognize any MHC in the population, these receptors are perfectly capable of detecting the presence (and hence absence) of MHC molecules within one individual. But if the KIR-MHC interaction is specific enough (i.e. 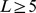), the chance of a KIR to recognize any MHC within the same individual is small, impeding the host to detect MHC down-regulation, i.e. missing-self. Thus, the potential to recognize the absence of MHC molecules, and with it to clear the infection, decreases with a higher specificity of KIR-MHC interactions. Note that the inability of a specific inhibitory KIR to recognize missing-self is independent of the education process we implemented in the model. These results were consistent in all simulations we ran for each specificity setting (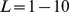, Fig. 2 E red line), confirming our reasoning that for missing-self detection, inhibitory NK cell receptors do not need to be specific.

### Specific inhibitory KIRs protect hosts against a virus evolving decoy molecules

To avoid elimination by the host immune response, viruses like CMV code decoy MHC molecules that can engage inhibitory NK cell receptors [26], [27]. As KIR specificity did not have a large effect on missing-self detection, we wondered whether high KIR specificity can be an adaptation to a CMV like virus. In our model, a virus down-regulating the MHC expression in one individual, can randomly select one of the MHC molecules of its host, incorporate it in its “genome”, and express it as a decoy protein in the current and subsequent hosts. While in the current host this decoy is always successful, in the subsequent hosts its success will depend on the specificity of the KIRs. Viruses carrying successful MHC decoys can escape the immune response of both T and NK cells. The fitness cost of a host infected with one of these successful viruses was modeled by decreasing the probability of clearing the infection to zero (Fig. 1 B). Thus, each individual with KIRs recognizing a foreign viral decoy like self MHC, became chronically infected in the model.

The better adaptation of a decoy virus compared to the MHC down-regulating virus was reflected in a higher fraction of chronically infected individuals and in a lower population size (Fig. 2 C). But, opposite to what we observed with the virus down-regulating MHC, the effect of KIR specificity was drastic. The average population size increased from 2500 individuals in a degenerate system to 4100 in a very specific system (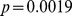, Mann-Whitney *U* test). Populations having specific KIR-MHC interactions were thus much better protected than those with degenerate or cross-reactive KIRs (Fig. 2 C, D, E).

Why is a highly specific KIR-MHC interaction advantageous in this CMV like infection? To protect the host, KIRs face the challenge to detect MHC down-regulation but not recognize the viral decoy masking MHC down-regulation. As seen in the previous section, a host with degenerate KIRs always has a large repertoire of licensed KIRs, and therefore always succeeds in detecting missing-self. But because of the same low specificity, the KIRs within that individual are expected to also recognize foreign decoy molecules as self MHC. On the other hand, a specific KIR system results in a smaller repertoire of licensed KIRs per individual, impeding the host's ability to detect missing-self (see Table 1 and previous section). However, because of their high specificity, it is also unlikely for any licensed KIRs to recognize foreign decoys. Therefore, a decoy virus typically fails to escape NK immune responses, allowing the infection to be cleared. Again, these results were consistent in all simulations we ran for each specificity setting (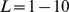, Fig. 2 E black line), showing that KIR specificity helps protecting individuals against viruses evolving MHC-like molecules.

### KIR diversity evolves as a consequence of high specificity

We next studied the effect of specificity on the evolution of KIRs. To estimate the diversity of KIR molecules in the population, we calculated the Simpon's Reciprocal Index (SRI) [33]. The SRI is a diversity measure that is equal to the total number of KIR alleles if they are all equally distributed in the population, whereas the SRI is lower than that in a population where some alleles dominate (described in Material and Methods). This measurement of diversity has the advantage that it is not sensitive to fluctuations in the frequencies of rare KIR alleles in the population.

**Figure 3:**
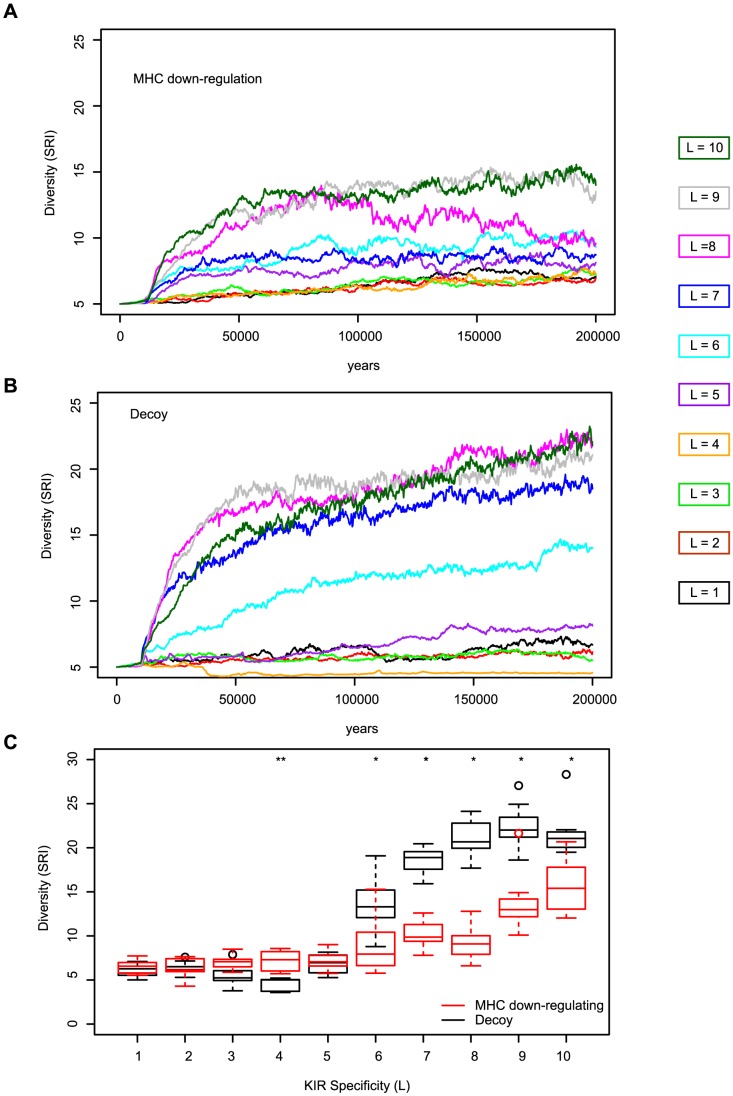
KIR polymorphism evolves only in populations having specific KIRs. The degree of KIR diversity (expressed as the Simspon's Reciprocal Index) increased only in populations having specific KIRs (i.e., for 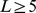), whereas in populations with degenerate KIRs it remained close to its initial value, reflecting largely neutral drift. The time plot in (A) represents the KIR diversity in a population infected with an MHC-downregulating virus, and the time plot in (B) shows the KIR diversity in populations infected with viruses evolving decoy molecules. Each line represents the mean of the SRI score, i.e. averaged over 10 different simulations. The different colors represent the specificity values ranging from 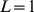 to 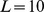 bits. (C) Mean SRI score at the end of the simulation for each specificity value. There is a significant difference in the SRI score between the infection with an MHC down-regulating virus (red) and an infection with a virus evolving decoy molecules (black) (* represent *p* values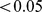 and ** *p* values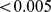, Mann-Whitney *U* test. The boxes represent the interquartile range, the thick horizontal lines represent the median, and the circles are outliers.).

KIR polymorphism remained low in populations having degenerate and cross-reactive KIRs (i.e. 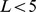), whereas it increased significantly in populations with specific KIR-MHC interactions (Fig. 3 A–B). Why is there only selection for diversity in those populations having specific KIRs? Since every host needs to recognize at least one of its MHC-molecules to have a licensed KIR repertoire, and this is guaranteed with degenerate KIRs, there is hardly any selection pressure in these populations to evolve novel KIR molecules. But with specific KIRs, individuals do not always recognize their own MHC and hence they are more vulnerable during the infection with an MHC down-regulating virus. The chance of recognizing self MHC is higher in individuals carrying two different haplotypes of inhibitory KIRs. Therefore, heterozygous hosts have an advantage over homozygous hosts, an effect that becomes larger with increasing KIR specificity. We conclude that this “heterozygous advantage” is the main selection pressure driving the evolution of novel KIR haplotypes, and that the selection pressure is largest in populations with specific KIR-MHC interactions.

### Specific and orthogonal KIR haplotypes emerge after infections with CMV-like viruses

We argued that heterozygous advantage drives the selection of novel KIR molecules in populations with specific KIR-MHC interactions (i.e. 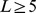). Yet, the KIR diversity differed significantly between simulations with MHC down-regulating and decoy viruses for exactly the same specificity (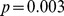, and 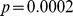, Mann-Whitney *U* test, Fig. 3 C). This result was surprising, because the heterozygous advantage in the likelihood of recognizing self MHC should be equally strong in both types of infection. To address the possible mechanisms underlying this result, we studied the KIR molecules that were being selected after an infection with the CMV-like virus.

**Figure 4:**
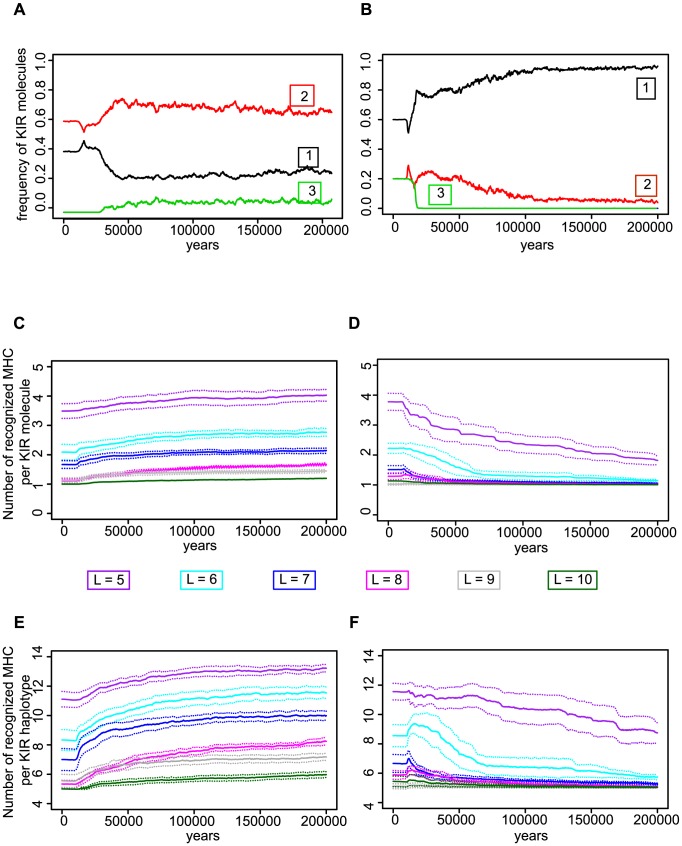
Viruses that down regulate MHC expression select for degenerate KIR molecules while viruses expressing decoy molecules drive the selection of specific receptors. (A)–(B) Single representative simulations in a population infected with highly specific KIRs (i.e. 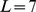 bits). (A) The number of MHC molecules recognized per receptor in a population infected with an MHC down-regulating virus. The initial KIR molecules happen to recognize different numbers of MHC molecules, as indicated by the numbers in the boxes. 40% of the KIR molecules recognized one MHC molecule (black line), and 60% recognized two MHC molecules (red line). Upon infection, new KIR receptors that happened to be more cross-reactive, i.e., recognizing three MHC molecules (green line) evolved, while the most specific KIRs decreased in frequency. (B) The specificity of the KIRs in a population infected with decoy viruses. 60% of the inital KIR molecules recognized one MHC molecule (black line), 20% recognized two MHC molecules (red line), and 20% happened to recognize three different MHC molecules (green line). Over time, the frequency of those KIR molecules recognizing only one MHC molecule increased and they take over the population. (C)–(D) The average number of MHC molecules recognized per KIR molecule. In populations with highly specific KIRs (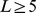 bits), this number decreases if the individuals are infected with a virus down-regulating MHC expression (C), whereas it increases in populations infected with decoy viruses (D). (E)–(F): The average number of MHC molecules recognized per haplotype. We observed a similar behaviour as in (C)–(D). The heavy lines represent the mean number of recognized MHC molecules over ten simulations. The different colors represent the specificity values ranging from 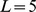 to 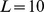 bits. The standard deviation is depicted by the dashed lines.

First, we analyzed the specificity of the KIR molecules. Although the specificity threshold 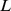 was fixed, some KIRs happened to recognize more MHC molecules than others. In populations with highly specific KIR-MHC interactions (e.g. 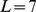), the initial haplotype was composed of 5 KIRs, each of them recognizing a different number of MHC in the population (Fig. 4 A,B). This distribution remained constant until the mutant viruses emerged. When the CMV-like virus was introduced, there was a clear selection for those KIR molecules that were most specific, i.e. KIRs recognizing only one MHC molecule in the population (Fig. 4 B). Similarly, populations infected with an MHC down-regulating virus evolved more cross-reactive KIR molecules (Fig. 4 A). Thus, although the specificity threshold 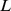 was fixed, the system exploited the stochastic variation in cross-reactivity among KIR molecules, evolving towards the specificity that rendered most protection (Fig. 4 C,D). During an infection with decoy viruses, this selection started already in populations having intermediate specific KIRs (i.e. 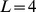). Surprisingly, a higher specificity was achieved by haplotypes implementing duplicate KIR genes, which effectively decreased the number of loci (Fig. S2). The fact that the evolution of an even higher specificity affects the heterozygote advantage (Fig. S1) explains the variation in KIR diversity between the infections with MHC down-regulating and decoy viruses (Text S-S).

If evolution selects for the most specific KIRs to protect against viruses evolving decoys, the challenge of recognizing self MHC is even larger. Is there any mechanism that allows for a higher chance of detecting self despite a high KIR specificity? To address this question, we studied the KIR haplotypes before and after the infection with a decoy virus, and again measured the number of recognized MHC per haplotype. In populations having specific KIRs (e.g. 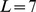), a randomly generated initial haplotype recognized an average of 8 MHC molecules, reflecting the expected cross-reactivity of its KIRs. Upon infection with decoy viruses, more specific haplotypes evolved due to the evolution of more specific KIR molecules. At the end of the simulation, approximately 80% of the haplotypes recognized only five different MHC molecules in the population (Fig. S3); a surprising result because this property is only expected for 45% of the randomly created KIR haplotypes with 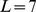. Hence, there was a clear selection for haplotypes that overlapped as little as possible in their MHC recognition, while keeping the highest specificity per KIR molecule. By evolving such “orthogonal” haplotypes, the paradox of recognizing as many MHC in the population as possible without detecting foreign decoy molecules was solved. Together, our analysis suggests that, if the specificity of KIR increases, it becomes beneficial to have more loci to be able to detect missing-self, which provides an explanation for the observed polygeny in the KIR complex.

## Discussion

The exact evolutionary advantage of the highly diverse KIRs has remained intriguing, especially because MHC class I detection, i.e. “missing-self” detection, can also be achieved by a limited set of monomorphic receptors. Our results show that for simple detection of MHC down-regulation, degenerate KIR molecules are advantageous, while a specific KIR-MHC interaction protects hosts against viruses evolving decoy molecules. In the presence of viruses expressing decoy molecules, the KIR became very specific, while at the same time the number of recognized MHC molecules per haplotype was maximized. The more specific the system becomes, the stronger the selection pressure on hosts to carry two different KIR haplotypes. As a result of this heterozygote advantage, KIR haplotypes evolve a high degree of diversity.

The results with viruses evolving decoy molecules depend strongly on the implemented MHC dependent NK education process, as we allow only for the “licensed” KIRs to participate in the immune response. Inhibitory receptors for MHC class I are very important for the education, repertoire development, and response of NK cells, and there is indeed good evidence that the failure to engage inhibitory receptors during development results in peripheral NK cells that are hyporesponsive [32], [34]–[37]. Yet, recent studies [38]–[40] showed that NK cell populations that cannot ligate their inhibitory receptors–either because they are unlicensed cells, or because they have been transferred into a different MHC class I environment–respond in a normal inflammatory manner after viral infection. This response of unlicensed NK cells appeared to be even more robust and protective than that of licensed NK cells. In all these studies, the NK cells were stimulated via their activating receptors. However, we only considered inhibitory receptors, modeling “functionality” as the capacity of the mature NK cells to respond to cells in which the expression of self MHC class I is decreased. MHC independent mechanisms for NK cell activation, such as activation via cytokines, is implicit in the model, and is taken into account in the overall probability of clearing the infection. Also note that we do not model the KIR molecules on individual NK cells, but define which KIRs are licensed in a host's whole repertoire.

The KIR system has evolved unusually rapidly, resulting in different levels of specificities across species. While KIRs in rhesus macaque have a broad specificity, orangutans, chimpanzees, and humans have evolved more specific KIR systems [41], [42]. KIR recognition in humans is restricted to at least four epitopes (HLA-A11,-Bw4,-C1, and -C2), where HLA-C1 and -C2 have the highest avidity. The evolution of these particular MHC epitopes have left an imprint on the evolution of the KIR system. This is clearly shown in the differences of the KIR haplotypes starting from old world monkeys to humans [43]. Humans, chimpanzees, bonobos, gorillas, orangutans, and rhesus macaques share four lineages of KIR genes, which expanded approximately 35–40 million years ago [31]. Within these lineages, each species has independently evolved different numbers of KIRs with either an inhibiting or activating function, and their emergence is related to the evolution of their ligands. The expansion of lineage III KIRs in orangutans, chimpanzees, and humans is associated with the emergence of the C1-, and later with the C2 epitopes. In contrast, rhesus macaques have expanded lineage II KIR genes, corresponding to their complex MHC system, which is composed of several subsets of differentially expressed MHC-A, and -B genes in the absence of an MHC-C locus. Here, we do not model the four KIR epitopes present on several MHC alleles, but have randomly made MHC molecules. The composition of KIR haplotypes, as well as the evolution of novel MHC molecules, is not the focus of this manuscript. Our main question is why KIRs have evolved a degree of specificity, and our approach clearly reveals that specificity has a selective advantage because of its protective effect against CMV-like viruses evolving MHC decoys. Some of the high specificity values used in our model might seem contradictory to the small number of MHC epitopes identified as main KIR ligands. Yet, all results presented here are already obtained at specificity values 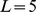, which corresponds to a recognition of 20% of MHC molecules in the population (see Table 1), and is in agreement with the four MHC epitopes that have been identified so far in human KIRs.

Hosts are exposed to multiple challenges during their life span, and the immune system has evolved to respond to all of them rather than adapt to only one particular virus. For simplicity, we here consider only one type of infection at a time. Nevertheless, CMV seems to have an important role in NK cell mediated immunity. Recent studies revealed that there is a strong imprint in the NK cell repertoire of CMV seropositive individuals because a particular subset of NK cells with “self-specific” inhibitory KIRs is expanded [29], [30]. Furthermore, it has been shown that CMV plays an important role in viral driven evolution of NK cell receptors in mice [44], [45]. Mice possess the Ly49 receptor system, which is functionally similar to KIR but evolutionary and structurally different. The Ly49 receptors exhibit also high genetic diversity and also have mouse MHC-class I molecules as ligands. Mouse strains that are resistant to MCMV carry an activating receptor, Ly49H, binding to the “MHC-class I decoy” m157 with high affinity. Mice susceptible to MCMV lack the Ly49H gene but possess the inhibiting receptor Ly49I also binding strongly to the m157 glycoprotein. Because Ly49H evolved from its inhibitory homologue, Ly49I [46], it seems that the m157 induced immune pressure led to the evolution of a new activating NK cell receptor, conferring resistance to the virus. Our results agree with this data, showing that a CMV encoded MHC-like decoy imposes a selection pressure to drive the evolution of novel NK cell receptors.

Fighting pathogens and successful reproduction are two crucial functions for survival. By their contribution to immune defense and reproduction, KIRs reveal various selection pressures imposed on NK cells, emphasizing the importance of diversity for surviving population bottlenecks and infections. For these reasons, it may seem intuitive that receptor diversity is beneficial for viral control. But we have seen that the mere detection of missing self is achieved best with degenerate KIRs.

Our agent-based model provides a solid explanation for one selection pressure driving the evolution of specific KIRs, namely viruses expressing MHC decoys. This does not need to be the only explanation, and our findings call for further studies into other possible mechanisms. The evolution of the specificity and number of loci per haplotype, as well as the evolution of activating receptors or other viral strategies, should now be integrated in our model to address additional questions.

## Materials and Methods

### Agent-based model

We developed an agent-based model consisting of two types of actors (hosts and pathogens) and three types of events (birth, death, and infection). The basic time step of the model is one week, during which we run through all hosts in a random order and confront them to one of the randomly chosen events. Hosts age over time, and after each time step, their age, infection state, and infection type is updated. The cycle is repeated for many hosts generations to model the long-term evolution. All model parameters are fully described in Table 2. The following is a detailed description of the actors and the events:

**Table 2:** Model parameters.

Parameter	Value
Time step	1 week
Simulation time	2000 centuries
**Host parameters**	
Maximal population size 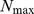	4980 individuals
MHC diversity	1 locus with 14 alleles
Number of KIR loci	5
Bit string length	16 bits^a^
Specificity 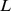 (bit scale)	1–10 bits
Host mutation rate 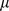	0.00005 per allele per birth event
**Infection** b	
Infection state 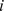	0 (susceptible), 1 (acute), 2 (chronic)
Probability of viral transmission during acute phase 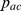	0.85 per contact
Probability of viral transmission during chronic phase 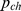	0.15 per contact
Probability of clearing the wild-type virus 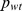	0.85
Probability of clearing the MHC downregulating virus 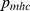	0.75
Probability of clearing the virus evolving decoy molecules 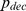	0
Immunity time 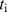	10 years
Acute infection time 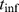	4 weeks
**Virus parameters**	
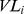	0 (for 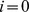 ), 0.1 (for 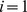 ), 0.06 (for 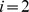 )
Virus mutation rate 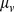	0.0001 per week
**Initial conditions**	
Initial population size 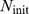	4500 individuals
KIR initial diversity (SRI)	5 (1 allele per locus)

a : The choice to use 16-bit strings represents a large enough theoretical repertoire of 65,536 sequences.

b : The parameters used for the infection are chosen to maintain the epidemic. Changing the length of the acute phase or the probabilites of clearance do not affect our results on the evolution of the KIRs qualitively (results not shown).

#### Hosts and viruses

Hosts are modeled as simplified humans with a diploid genome encoding for one MHC class I locus and one KIR haplotype composed of five different inhibitory genes. For simplicity, we only model one MHC locus with 14 different possible alleles. KIR and their ligands are represented as bit strings, as a simplified representation of amino acid sequences [47]. The simplest way to think about this representation is to see the proteins as being composed of two types of amino acids, e.g. hydrophobic, and hydrophylic. Interactions between two molecules are only allowed if the complementary matches between the strings exceed a predefined threshold. Since two molecules may interact in more than one way, the strings are allowed to match in any possible alignment. In our model we use a predefined threshold 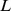, i.e. if the length of the longest adjacent complementary match between a ligand and a KIR is at least 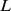 bits long, we consider the ligand to be capable of binding to its receptor (Fig. 1 A).

In our simulations, we consider in total 16 viruses: a wild type virus, one MHC down-regulating virus, and 14 decoy viruses. Each virus comes with a particular increase in the natural intrinsic death rate of its host 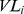, and a probability of clearing the infection 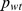, 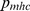, and 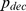. To escape an assumed T-cell response, the wild type virus evolves MHC down-regulation with a mutation rate 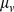. The decoy viruses evolve, in addition to MHC downregulation, a decoy protein mimicking an MHC molecule. We model the evolution of decoy molecules by allowing the virus to adopt a randomly selected MHC molecule from its host with the same rate 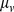. We do not allow for viruses to accumulate these decoy proteins: when viruses loose the previous decoy, they can adopt a new MHC molecule. Because we fix the polymorphism of the MHC molecules to 14 alleles, the maximal number of decoy proteins that can evolve in the population is 14. We also do not allow for superinfection and hosts can be infected with one of the 16 viruses only. The immune escape of the mutant viruses is modeled by decreasing the initial probability of clearing the infection from 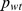 to 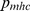, and 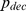 (Fig. 1 B, and Table 2).

The wild type virus is introduced once the population dynamics have reached a steady state. Similarly, the viral mutants are allowed to evolve 5000 years after introduction of the wild type virus.

#### Birth

We consider sexual reproduction, where each selected host passes one of its KIR haplotypes to its offspring. To maintain the initial polymorphism and the relative frequency of MHC alleles in the host population, we let the offspring inherit one MHC gene from one randomly selected parent while the other one is drawn out of a predefined pool of MHC alleles (see Model Initialization). The probability of a birth event taking place is described by 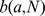 and decreases linearly with the population size (i.e. we consider logistic growth). Additionally, hosts with age between 20 and 45 are considered to be more fertile than children and the elderly, and at low population sizes fertile individuals are expected to give birth every two years. The resulting yearly birth rate (shown in Fig. S4 A) is described by:
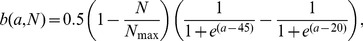
(1)where 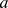 is the age of the parent, 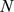 the actual population size, and 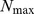 maximal population size.

KIR genes undergo mutation with a probability 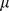. To decrease computation time we model mutations by randomly drawing a new KIR gene out of a large pool of predefined KIR alleles. Other mutational operators, e.g. point mutations, recombination, deletion or duplication are not considered here. The NK cell education process takes place at birth, where only those KIRs in the newborn that recognize at least one of its own MHC molecules, are set to be licensed. Hosts without any licensed KIR can be born, but they will be unable to detect MHC down-regulation. Newborn children are given the age of one and are added to the host population.

#### Death

A death event takes place with an age and infection-dependent rate 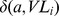. The yearly intrinsic death rate is a mathematical approximation of a human age-specific mortality curve [48]. If the host is infected with a virus, the death rate is increased by a factor 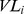, representing the effect of viral load during the different infection stages 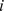. The resulting death rate (Fig. S4 B) is described by:
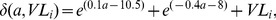
(2)where
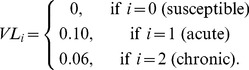
(3)


#### Infection

During each time step we challenge all susceptible hosts with possible infections by allowing every susceptible individual to contact one randomly selected partner. If this partner is infected, it will transmit its virus with a probability 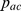 or 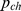 depending on the infection state of the infecting host (see Table 2). Properties of the virus, i.e. its evasion strategies, are not changed upon transmission.

After transmission, every individual has an acute infection for a period of 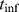 weeks, during which the intrinsic death rate is increased by 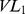 (see eq. 2 and eq. 3). After the initial acute phase the virus can be cleared with a probability 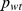, 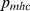, or 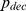, depending on the type of virus. If the individual fails to clear the infection, it will become chronically infected, and the death rate will decrease to 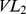. Recovered individuals are resistant to the virus, losing their immunity only after a long period, 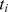.

Whether a virus can spread through the population depends on the number of newly infected cases it can generate during the course of its infectious period (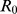). In our model, the number of new cases depends on the transmission probabilities, and the infection state of the host. Because the acute phase is relatively short (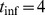 weeks long) and a chronic infection lasts for several years (until the infected host dies), viruses inducing a chronic infection, spread much better through the population. Therefore, the probability of escaping the acute immune response (i.e. 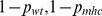, and 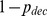) plays a major role in the 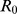 of the virus.

### Model initialization

The model was initialized with a host population of 4500 hosts, with a random age between 1 and 70 years. Gene pools for MHC and KIR alleles were created at the start of each simulation. The pool of MHC consisted of 14 alleles according to the most frequent HLA-C alleles in the European population (dbMHC Anthropology [49]). For each MHC allele, ten different KIR were randomly generated, which could bind to the MHC with a specificity of at least 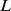, resulting in a KIR pool of “functional” 140 alleles. To create the initial genome of each host, MHC and KIR genes were randomly drawn from the pools. The individuals were initialized with the same KIR haplotype, but with different MHC genes.

### Genetic diversity

The Simpson's Index is a measurement of diversity that can be interpreted as the probability that two randomly chosen molecules from two random hosts in the population are identical. The lower the Simpson's Index, the higher is the diversity of molecules in the population, and the reciprocal of the Simpson's Index [33] defines a “weigthed” diversity. This diversity measure has the advantage over the total number of unique KIR molecules because it is less sensitive to fluctuations in molecule numbers caused by random neutral drift. For instance, if all molecules are equally frequent in a population, the SRI score is equal to the number of alleles in the population. A population dominated by a single molecule will have an SRI score close to 1. The SRI was calculated as follows: 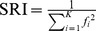, where 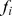 is the fraction of the molecule 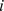 over all KIR molecules in the population, and 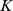 is the total number of unique KIR molecules.

### Analytical expectations

The probability that a host having a heterozygous diploid genome recognizes its own MHC molecules is defined by 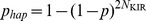, where 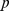 is the probability that a KIR recognizes a random MHC molecule in the population (which depends on 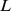, see Table 1), and 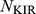 is the number of KIR loci. The expected number of licensed KIR is determined by 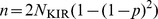. In our model each host has a genome consisting of one MHC locus and five KIR loci, i.e. 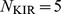, hence that individual will recognize its own MHC molecules with a chance 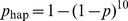, and the expected number of licensed KIR for the same individual will be 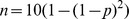. The expected protection against a decoy virus, i.e. the probability of not recognizing the viral protein as self MHC molecule, depends on the size of the licensed KIR repertoire, 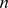, and is described by 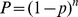.

Heterozygous advantage is defined as: 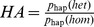, where 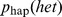 and 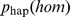 represent the probability of recognizing self MHC molecules for a heterozygote and a homozygote individual, respectively. We obtained 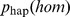 by measuring the fraction of MHC molecules detected by a single KIR haplotype. To obtain 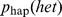 we measured the fraction of recognized MHC by all pairwise combinations of KIR-haplotypes. The population has heterozygote advantage if 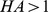. Values of HA for different 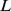, 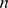, and 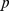 are given in Table 1.

### Implementation

The model was implemented in the C++ programming language. The value of 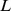 was varied in a range from one to ten. For each value 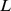, ten simulations were performed for 2000 centuries.

## Supporting Information

Figure S1
**Heterozygous advantage is higher in populations infected with a virus evolving decoys.** (A) Probability of recognizing a randomly selected MHC molecule per KIR haplotype, as a function of the KIR specificity 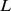. The observed values after infection with an MHC down-regulating virus (blue line) or viruses evolving decoy molecules (red line) differ from the expected probability value (black line). (B) Heterozygous advantage (i.e. 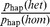 , see Material and Methods) as a function of the specificity 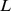. KIR evolve to become more degenerate after infection with an MHC-downregulating virus, therefore the selection pressure (blue line) is lower than expected (black line). In contrast, KIR molecules evolve a higher specificity upon infection with a virus evolving decoys. Hence, heterozygous advantage is higher in populations infected with a virus evolving decoy molecules (red line). Data represent the mean out of ten simulations per specificity value.(EPS)

Figure S2
**KIR haplotypes decrease their number of loci.** Upon infection with a virus expressing decoy proteins (occurring at 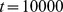), the initial haplotype composed of five unique KIR molecules (blue line) decreases in its frequency. The original haplotype is taken over by a new haplotype having only four unique KIRs, i.e., one KIR is a “duplicate”, which is indeed a strategy to become more specific. Single representative simulation of a population with intermediate specific KIRs (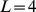 bits).(EPS)

Figure S3
**Viruses expressing decoy molecules drive the selection of specific “orthogonal” haplotypes.** The time plot depicts a single representative simulation of a population with highly specific KIRs (i.e. 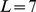 bits). The initial KIR haplotype happened to recognize exactly seven different MHC molecules. Upon infection with a virus expressing decoy proteins, new haplotypes recognizing different number of MHC molecules emerged. Haplotypes recognizing fewer MHC alleles increased over time and in this example the population acquired haplotypes covering five or seven MHC molecules.(EPS)

Figure S4
**Birth and death rate of the model.** (A) The age-dependent birth rate of a host population. The different lines show how the birth rate changes with different population size 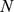. (B) The different lines depict the influence of the viral load 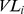 on the intrinsic age-dependent death rate of a host population.(EPS)

Text S1 **Heterozygous advantage is higher in populations infected with viruses evolving decoy proteins.**
(PDF)

Text S2 **Haplotypes with maximal MHC coverage limit heterozygous advantage.**
(PDF)
