# Supplementary material for: Virus Encoded MHC-Like Decoys Diversify the Inhibitory KIR Repertoire
Source: PLoS Comput Biol. 2013 Oct 10;9(10):e1003264. doi: 10.1371/journal.pcbi.1003264 (PMC3794908; doi:10.1371/journal.pcbi.1003264)
Supplement: Text S1 — Heterozygous advantage is higher in populations infected with viruses evolving decoy proteins. (PDF) [file pcbi.1003264.s005.pdf]

Text S1: Heterozygous advantage is higher in populations infected with viruses evolving decoy proteins

We measured significant differences in the SRI score between the viral strategies when KIRs are specific (i.e. $L \geq 5$), and for one intermediate specificity (i.e. $L = 4$). This observation strongly suggests that the different viral evasion routes impose different selection pressures. To study how this comes about, we first measured the fraction of MHC molecules recognized per haplotype (Fig. S1 A), and calculated the advantage of heterozygotes over homozygotes in the detection of MHC molecules (Fig. S1 B).

The observed haplotype specificity and heterozygous advantage confirmed our analytical expectations only in degenerate KIR systems, whereas, in specific systems, haplotypes evolved a different specificity than the threshold L (Fig. S1 A). In response to the MHC-downregulating virus, KIR haplotypes recognizing more MHC molecules were selected. But during an infection with the decoy viruses, KIR haplotypes recognizing less MHC molecules dominated the population. This is in agreement with our previous observations that more cross-reactive KIRs evolve after an infection with an MHC down-regulating virus, and more specific KIRs evolve during an infection with a decoy virus.

Given that the heterozygous advantage becomes larger with increasing specificity (Fig. S1 B), it is natural that the level of diversity is higher in populations infected with decoy viruses than in populations infected with an MHC down-regulating virus. Summarizing, these results show that the actual heterozygous advantage depends on the specificity of the KIRs, which can be higher or lower depending on the virus type the population is infected with.

The observed significant difference in the SRI in intermediate specificity $L = 4$ arises from yet another strategy. These systems evolved to become more specific by incorporating KIR genes as duplicates within the same haplotype (Fig. S2). By decreasing the number of unique KIR genes per haplotype, the number of recognized MHC molecules per haplotype is lower, and the overall probability of recognizing the decoy decreases.
