# Supplementary material for: Virus Encoded MHC-Like Decoys Diversify the Inhibitory KIR Repertoire
Source: PLoS Comput Biol. 2013 Oct 10;9(10):e1003264. doi: 10.1371/journal.pcbi.1003264 (PMC3794908; doi:10.1371/journal.pcbi.1003264)
Supplement: Text S2 — Haplotypes with maximal MHC coverage limit heterozygous advantage. (PDF) [file pcbi.1003264.s006.pdf]

Text S2: Haplotypes with maximal MHC coverage limit heterozygous advantage

The evolved orthogonal haplotypes have a strong effect on the heterozygous advantage (Fig. S1 B, red line). In our model, a single orthogonal haplotype recognizes exactly five different MHC molecules because it is composed of highly specific KIR molecules recognizing only one of the 14 MHC in the population.

A single haplotype is expected to detect 5 out of 14 of the MHC molecules in the population (Fig. S3). Consequently, the advantage that a heterozygous host carrying these complementary haplotypes has over a homozygote is smaller than the heterozygous advantage of host carrying non orthogonal haplotypes. Hence, the selection pressure reduces somewhat in orthogonal cases, and it reaches a plateau at $HA = 1 - \frac{(1-0.4)^2}{0.4} = 1.65$.
